# Use of Cellulose Nanofibers as an Electrode Binder for Lithium Ion Battery Screen Printing on a Paper Separator

**DOI:** 10.3390/nano8120982

**Published:** 2018-11-27

**Authors:** Oussama El Baradai, Davide Beneventi, Fannie Alloin, Yann Bultel, Didier Chaussy

**Affiliations:** 1 Univ. Grenoble Alpes, CNRS, Grenoble INP, LGP2, 38000 Grenoble, France; ussielba@gmail.com (O.E.B.); didier.chaussy@pagora.grenoble-inp.fr (D.C.); 2 Univ. Grenoble Alpes, Univ. Savoie Mont Blanc, CNRS, Grenoble INP, LEPMI, 38000 Grenoble, France; fannie.alloin@lepmi.grenoble-inp.fr (F.A.); Yann.bultel@lepmi.grenoble-inp.fr (Y.B.)

**Keywords:** cellulose nanofibers, Li-ion battery, printing electrode

## Abstract

Water-based inks were formulated using cellulose nanofibers as a binder in order to directly front/reverse print lithium ion cells on a paper separator. Moreover, the high cohesion of electrodes as provided by cellulose nanofibers allowed for the embedding metallic current collectors in the electrodes during the printing stage, in order to develop a one-step printing and assembling process. Positive and negative inks based on LiFePO_4_, or graphite, respectively, and cellulose nanofibers, displayed rheological properties complying with a variety of printing processes, as well as with screen printing. Printed cells exhibited high electrical conductivity and adhesion between current collectors and inks, i.e., up to 64 ± 1 J/m^2^. Electrochemical cycling tests at C/10 showed a reversible capacity during the first cycle of about 80 mAh/g, which slightly decayed upon cycling. Preliminary results and assembling strategies can be considered as promising, and they represent a quick solution for the manufacturing of lithium ion batteries. Work is in progress to improve these processing issues and the cycling performances of Li-ion cells.

## 1. Introduction

Because of its large availability and excellent mechanical properties, over the last 10 years, cellulose and its derivatives have been widely used for the fabrication of composite materials with widespread applications ranging from structural materials, where fibers are used as reinforcing elements in both synthetic and bio-sourced polymeric matrices [1], to semi-permeable membranes [2,3] and functional substrates/binders for printed electronics [4]. In parallel to composite material designs, progress in cellulose nanocomposites processing [5] is paving the way for their industrial use. Among the application fields of cellulose nanofibers, energy storage is attracting an ever increasing level of interest, since cellulose in the form of fibers, nanofibers, and water-soluble polymer (i.e., carboxymethyl cellulose) has been successfully used in lithium ion batteries (LIBs) for the fabrication of electrodes, separators, reinforced gel-, or dried polymer–electrolytes [6,7,8]. The use of cellulose derivatives allows for a “greener” form of processing, since the ink formulation does not require the use of volatile and toxic organic solvents. Moreover, several authors have formulated electrode inks using cellulose derivatives as binders, i.e., methyl-, ethyl-, carboxymethyl-, and microfibrillated-cellulose [9,10,11,12], and have shown that cellulosic binders can provide electrochemical and cycling stability that is equivalent to those obtained using conventional fluorinated binders and organic solvents (i.e., polyvinylidene fluoride, PVdF, and *N*-methyl-2-pyrrolidone, NMP). Concerning the manufacturing process, printing processes have been selected as a very economic production method, and they play a more and more important role in LIBs manufacturing strategies [13,14]. Indeed, contrary to the coating method, printing techniques allow for the elaboration of patterns on different substrates with minimal extra cost production. The morphological and electrochemical properties of printed films strongly depend on the viscosity and the composition of the ink, which may be adapted to the process. Indeed, some studies have reported that the homogeneous dispersion of active particles in both solvent-based and aqueous slurries improves the electrode discharge capacity [15] and the cyclability/rate capability [16,17].

In addition to optimal ink formulation and dispersion, the stacking and the geometry of cell components has a crucial role in determining the performance of the complete electrochemical cell. In terms of packaging solutions, flexible assembling designs have been proposed in the last few years to overcome limitations due to mechanical stresses and shape requirements in LIBs manufacturing processes [18,19,20]. However, despite the production of thin-flexible, bendable, origami foldable batteries, the assembly of the base electrochemical cell relies on its sequential deposition on a mechanically stable substrate of: (i) a metallic current collector; (ii) the electrode material, a separator/electrolyte. The counter electrode and the associated current collector can be further deposed on the separator or mechanically laminated. Leaving aside the packaging stage, up to four to five printing/lamination stages can be made necessary for the full cell assembly.

This work shows preliminary promising results for an LIB where positive and negative electrodes are formulated by replacing conventional fluorine based binder (PVdF) with bio-sourced cellulose derivatives as nanocellulose, and carboxymethyl cellulose (CMC) and are screen-printed onto the front/reverse sides of a paper separator. Moreover, the high electrode cohesion provided by cellulose nanofibers allows for the embedding of metallic current collectors within the electrode material during the printing process, thus reducing the whole Li-ion cell assembly to a two-stage recto/verso printing sequence.

## 2. Experimental

### 2.1. Materials

Battery-grade graphite (GP) (Timcal^®^ SLP10, Bodio, Switzerland) and commercial lithium iron phosphate (LFP Prayon, Engis, Belgium), with a measured mean diameter of 9 µm and 100 nm, respectively, were used as active material for the fabrication of negative and positive electrodes. Carbon black (CB) and carboxymethyl cellulose (CMC) with an average molecular weight of 90,000 g mol^−1^ and a degree of substitution of 0.7 were purchased by the Rubber team^®^ (Bristol, UK) and Aldrich, respectively. Cellulose nanofibers (CNF) obtained from bleached Domsjö pulp and with a diameter and length of below 0.5 and 100 µm, respectively, and a carboxylic group content of 0.1 mmol/g, were purchased from “Institute Technologique Foret Cellulose Bois-construction Ameublement” (FCBA, Grenoble, France) in the form of a 2% (*w*/*w*) suspension. The CNF hydrogel was obtained using a mechanoenzymatic protocol with five homogenization stages at high pressure, i.e., 500, 1000, and 1500 bars [21]. The cellulosic-based substrate (i.e., a cell separator) was provided by Papyrus^®^, and it exhibited a thickness of 142 µm, an air permeability of 417 cm^3^·cm^−2^·min^−1^, and a porosity of 47%. These characteristics ensure electrical isolation between the electrodes, and simultaneously guarantee a ionic conductivity of 3 mS/cm at 20 °C when swelled by a 1:1 volume mixture of ethylene carbonate (EC), dimethylcarbonate (DMC), and 1 M (LiPF_6_) (battery grade provided by Aldrich. Copper and aluminum grids (Alfa Aesar^®^,Haverhill, MA, USA) were used as current collectors. The current collectors have a thickness of 50 µm and a porosity of 72%, with a pore area of 0.16 mm^2^.

### 2.2. Ink Formulation

**Figure 1:**
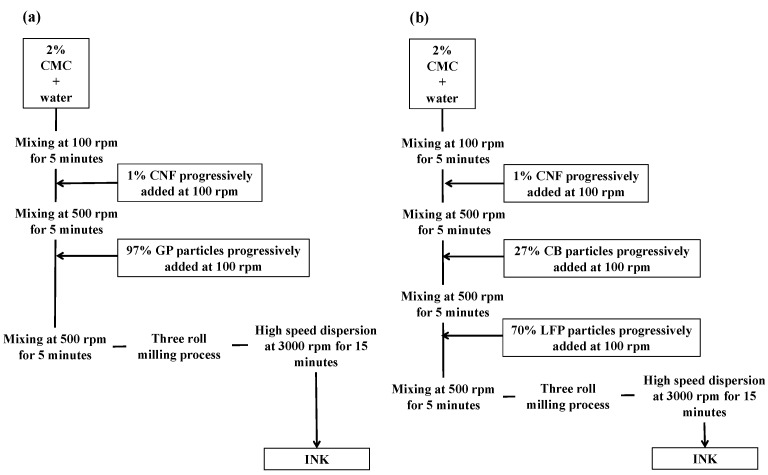
Flow chart of the formulation protocols used for the preparation of (**a**) negative and (**b**) positive electrodes.

Electrode inks were prepared by following two similar protocols. All percentage weights are expressed with respect to the dry solid content (M_s_) that was fixed at 40% (*w*/*w*) for both inks. The formulation protocols are shown in Figure 1a,b.

For the negative electrode, CMC polymeric dispersant was first added in distilled water solvent. The obtained mixture was dispersed by a mechanical stirrer (VMA^®^ disperser) at a rotation rate of 500 rpm for 5 min. Secondly, mixture was gently mixed at 100 rpm, and the CNF binder was progressively added. Another mixing step at 500 rpm for 5 min was necessary to insure the good dispersion of the mixture. Graphite active particles were added at 100 rpm, and the prepared ink was further stirred at 500 rpm for 10 min. The ink was then homogenized by three rolls mill (provided by EXAKT^®^, Oklahoma City, OK 73116, USA), and the roll milled ink was finally dispersed at 3000 rpm for 5 min using high-speed disperser mixers.

For the positive electrode, CMC and CNF were added in distilled water as the first steps, as well as for the anode formulation protocol. The CB conducting agent was then added progressively to the CMC-CNF mixture, and mixed at 500 rpm for 5 min (VMA^®^ disperser, Reichshof, Germany). Finally, the LFP powder was added to the suspension and the system was stirred for 5 min at 500 rpm. The ink was then homogenized by a three rolls mill (provided by EXAKT^®^, Oklahoma City, OK 73116, USA), and the roll milled ink was finally dispersed at 3000 rpm for 15 min, using high-speed disperser mixers (Figure 1b).

Rheological properties of the inks were measured by using a MCR 301 rotational rheometer provided by Anton Paar^®^ (Graz, Austria) with a plate–plate geometry. Measurements were performed at 25 °C at a fixed gap of 1 mm. Steady-state flow measurements were carried on from 10^−1^ to 10^3^ s^−1^.

### 2.3. Printing Process

Cells were printed onto the cellulosic-based substrate by using a DEK Horizon 03i automatic flatbed press screen printing machine. A polyamide nylon screen mesh (208 threads per inch, 40% open area, 70 µm thread diameter, and 110 µm emulsion thickness) was used to achieve the printing tests. The printing speed was fixed at 30 mm/s, and with a printing force of 50 N.

The printed electrodes were dried first at 110 °C for 10 min, and secondly at room temperature overnight. Furthermore in order to remove water traces, electrodes were dried at 105 °C under a vacuum overnight, and store in a glove box. Cells were assembled in an Ar glove box into aluminum-laminated pouch cells.

### 2.4. Physical and Electrical Tests

The surface morphology was observed by using field emission scanning electron microscopy (FESEM, Zeiss Ultra 55) after sample deposition onto a carbon tape, and metallization under vacuum (AuPd). The electrical conductivity was measured using a four-probe ohm meter (Jandel Universal^®^ probe System with RM3-AR Test Unit). An adhesion test was performed by the means of a peel tester provided by Thwing Albert^®^ (Berlin, Germany). The measurement allows for the quantification of the force required to peel off a pressure-sensitive tape from a test panel at an angle of 180° degrees, and at a standard speed of 5 mm/s.

### 2.5. Electrochemical Test

The electrochemical behavior of the cell was tested at ambient temperature using an Arbin Testing Systems S/N 170795. Galvanostatic charge–discharge cycling tests were performed. The cell was discharged and charged at a constant current rate of C/10 (i.e., 17 mA/g).

## 3. Results and Discussions

### 3.1. Assembling Strategy of the Cell

**Figure 2:**
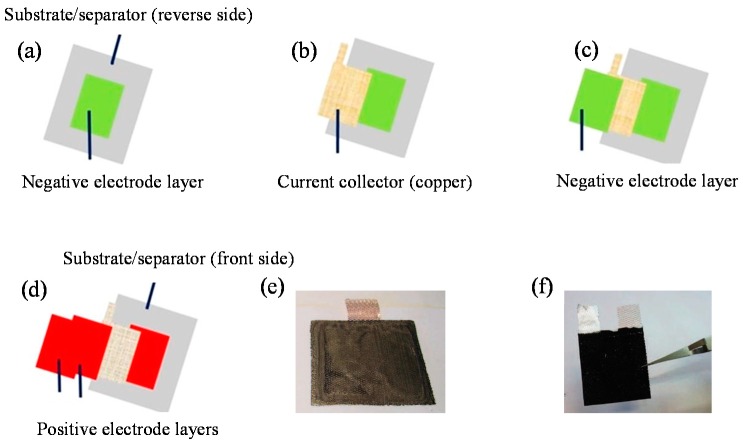
Schematic diagram showing the fabrication of the front/reverse assembled battery. (**a**) The negative electrode is printed onto the reverse side of the substrate; (**b**) the current collector is integrated in the structure, and (**c**) fixed with another printed layer. (**d**) On the front side of the substrate, the positive electrode is printed, but this time, the number of printed layers is modulated in order to match the capacity of the electrode. (**e**) Example of the electrode after the embedding of the current collector. (**f**) As a last step, the battery is assembled, with positive and negative components printed onto the front and reverse sides of the substrate.

The assembling strategy proposed in this work allows for the reduction of the number of printed layers, in order to improve the performance. In fact, the cellulosic membrane has, at the same time, the role of the substrate for the printing step, and a separator between electrodes. Moreover, contrary to a planar layout, it ensures a reduced mean path for the lithium ions during cycling [22]. Furthermore, current collectors, in the form of metal grids, have been integrated during the printing stage, in order to have a one-step printing and assembling process. As illustrated in Figure 2, the battery assembling strategy includes different steps. Firstly, one layer of negative material is printed onto the reverse side of the substrate (Figure 2a). Secondly, a copper current collector is placed onto the printed pattern, and another layer of the negative electrode is printed, in order to definitely fix the current collector (Figure 2b,c). On the front side of the substrate, the same principle is applied for the positive electrode.

An aluminum-based current collector has been used, and the number of printed layers required to fix the current collector was tuned, in order to match the capacity between electrodes (Figure 2d,e). Once the battery was assembled (Figure 2f), a packaging step was performed in a polymer-coated aluminum bag after the incorporation of the electrolyte. The electrolyte was incorporated by injection into the assembled cell just before sealing. The dimensions of the pouch cell were 140 × 90 mm.

### 3.2. Formulation and Rheological Characterization of the Inks

Before battery assembling, inks were formulated and printed. For the negative electrode, a graphite powder (GP) was selected because of its good performance and easy recycling properties [23]. CNF was used as a binder, and carboxymethyl cellulose (CMC) as a dispersing agent. CMCs are classically used to avoid the flocculation phenomena of the CNFs during the printing process [24]. For the positive electrode, a lithium iron phosphate (LFP) was selected because of its high performance and low environmental impact [25]. Carbon black (CB) was added as an electron conductor, in order to promote the electrical conductivity. The contents of the dry solid and its components were tuned to satisfy the rheological parameters for the screen printing process. In fact, printing processes require some restrictions in terms of ink viscosity, to have an appreciable print quality [26].

**Figure 3:**
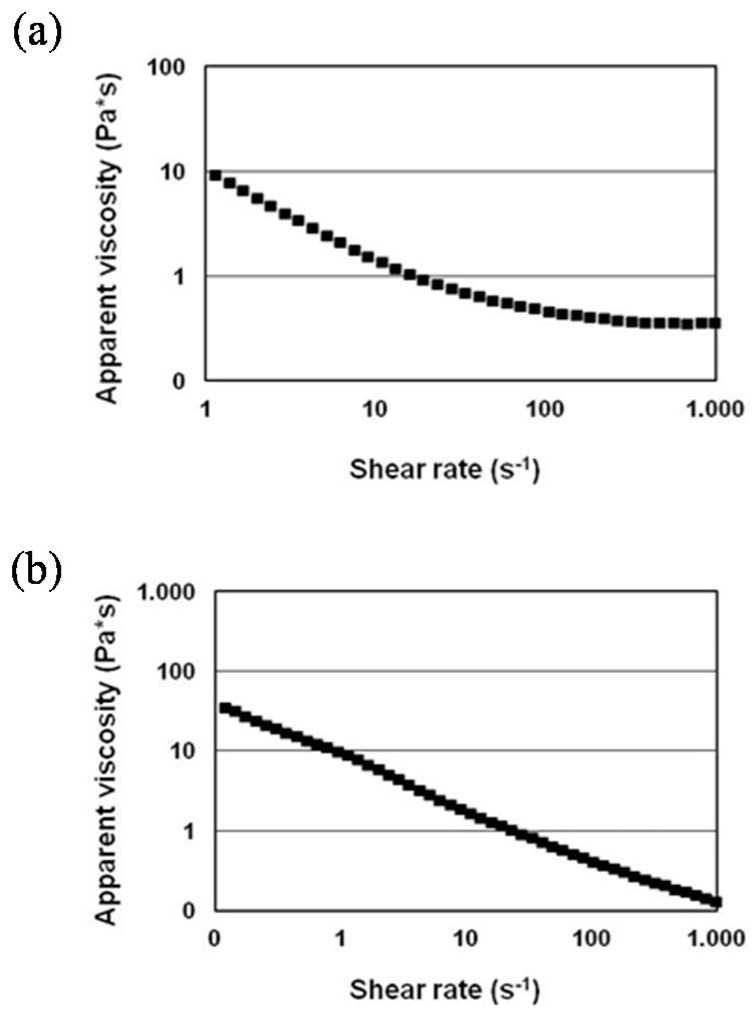
Rheological properties of the inks. Apparent viscosity is expressed as a function of shear rate for (**a**) negative electrode ink and (**b**) positive electrode ink.

The ink for the negative electrode was formulated by mixing GP, CMC, and CNF in distilled water in a 97:2:1 mass ratio, in order to obtain a dry solid content of 40% (*w*/*w*). The ratio of the components was tuned to match screen printing rheological requirements. CMC and CNF were fixed at 2% (*w*/*w*) and 1% (*w*/*w*), respectively, in order to avoid too high a viscosity due to the thickening effect of the CNF [27], or too low a viscosity caused by the dispersing effect of CMC [28]. The same principle was followed for the formulation of the positive ink by mixing LFP, CB, CMC, and CNF in a mass ratio of 70:27:2:1. The water content was adjusted to have a dry solid content of 40% (*w*/*w*), having the appropriate rheological properties for the process. Moreover, the content of CB (27% (*w*/*w*)) was higher than the measured percolation threshold, to ensure high electrical conductivity. A homogenization and dispersion protocol was developed by the means of a high disperser and a three-roll mill. Rheological investigation of inks was performed. As shown in Figure 3, inks exhibit a shear thinning behavior, with a decrease of the apparent viscosity when the shear rate increases. Indeed, screen printed inks should provide rapid switching in viscosity, with respect to the shear stress, in order to simplify the printing process, and to avoid too high a slump effect, or non-uniform coating. The measured viscosity values at low shear rates were conformed with screen printing process viscosity requirements ranging between 10 and 150 Pa·s^−1^ [26] at a shear rate corresponding to 1 s^−1^.

### 3.3. Physical Characterization of the Electrodes

Inks were screen-printed onto a cellulosic separator with a high grammage, conventionally used in the printing process. This separator insures good printing properties, thanks to the optimal coating layer pore structure that enhances the ink setting [29].

Inks were characterized from a physical and electrical point of view. Table 1 summarizes the main properties of the substrate, and the positive and negative printed electrodes. The addition of a large amount of carbon black inside the positive electrode provides an appreciable electrical conductivity of 37 S/m, which is higher than classical-casted electrode (2.5 S/m at 3.5% (*w*/*w*) of CB) [30]. The porosity of the electrodes was estimated by considering the density of each component. Porosities around 70% were calculated for both negative and positive electrodes. These values are comparable with the porosities of the screen-printed electrodes containing PVdF binder and organic solvent before the calendaring process [31]. However, they are higher than the value of 30%, reported as the optimum for high performances for conventionally manufactured electrodes after calendaring [32]. The calendaring process was not considered in these preliminary investigations, in order to avoid local short circuits due to the contact between the positive and negative electrodes promoted by CB migration in the cell.

The loading of the positive electrode must be high, in order to balance the capacity of the negative electrode. Moreover a part of the lithium ions intercalated in LFP is sacrificed to form the solid electrolyte interphase (SEI) layer at the negative electrode during the first charge [33]. Unfortunately, the deposition of a thick electrode induces its delamination. For this reason, the loading of the positive electrode was fixed at 70 g/m^2^ (which corresponds to a capacity ratio of P/N = 0.9), and thus, the capacity of the cell was limited by the capacity of the positive electrode.

**Figure 4:**
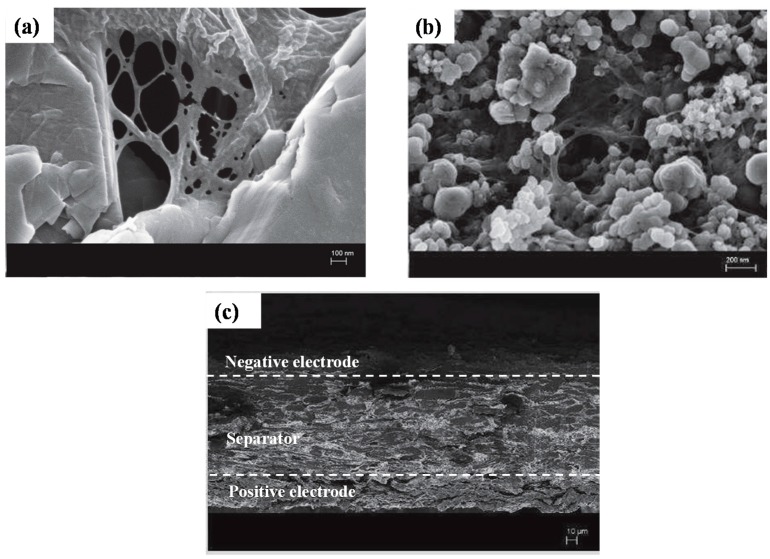
Field emission scanning electron microscopy (FESEM) images of surface electrodes and the printed battery. (**a**) Network structure between the graphite (GP) platelets and cellulose nanofibers (CNF) (mag: 50,000 ×, beam acceleration voltage: 3 kV, working distance 4.1 mm). (**b**) The same network structure has been observed for positive electrodes between lithium iron phosphate (LFP)/carbon black (CB) particles and the CNF network (mag: 20,000 ×, beam acceleration voltage: 3 kV, working distanceWD: 4.1 mm). (**c**) The cross-section of the full printed battery is showed with a three-layer structure corresponding to the separator and the positive and negative electrodes, and delaminations between the separator and the positive electrode are displayed (mag: 200×, beam acceleration voltage: 3 kV, working distanceWD: 4.2 mm).

SEM photos show the morphology of the composite electrodes. CNFs form a nanofiber network structure that may enhance the mechanical properties of the electrodes (Figure 4a). As an example in Figure 4b, LFP particles (blue arrow) are embedded in a CNF network (red arrow). Figure 4c shows the cross-section of the full-printed battery before the integration of the current collectors. Three different layers are evident. The top one represents the negatively printed electrode, and the bottom one the positive electrode. In the middle, the thick and tortuous cellulose-based separator insures electrical insulation between the electrodes. If we focus on the interface between the separator and the positive electrode, delamination defects are evident, which may be due to the higher material loading compared with the negative electrode.

As explained in Section 3.1, the key point of the front/reverse technique is the embedding of the current collectors in the electrode structure. In order to quantify the cohesive energy of the multilayer structure, a peel test was performed. This test allows for the quantifying of the energy required to remove the copper and aluminum current collector from the respective electrodes.

The adhesion energies required to peel off the current collectors from the positive and negative electrode layer are 64 ± 1 J/m^2^ and 23 ± 3 J/m^2^ respectively. The difference between the two values might be related to the CB content in the positive ink. In fact, as reported by Chen et al. [34], CB ensures the mechanical integrity of the electrode, thanks to its fractal structure by improving the adhesion forces. Higher tensile properties of CB than GP were also reported for composites with a carbon mass >5% [35,36], and they were associated with the low particle/matrix adhesion provided by laminar fillers (i.e., graphite sheets). Moreover the low adhesion energy of 12 J/m^2^ reported for conventional PVdF binder-based electrodes highlighted the role of CNF in increasing electrode mechanical properties.

### 3.4. Electrochemical Characterization

**Figure 5:**
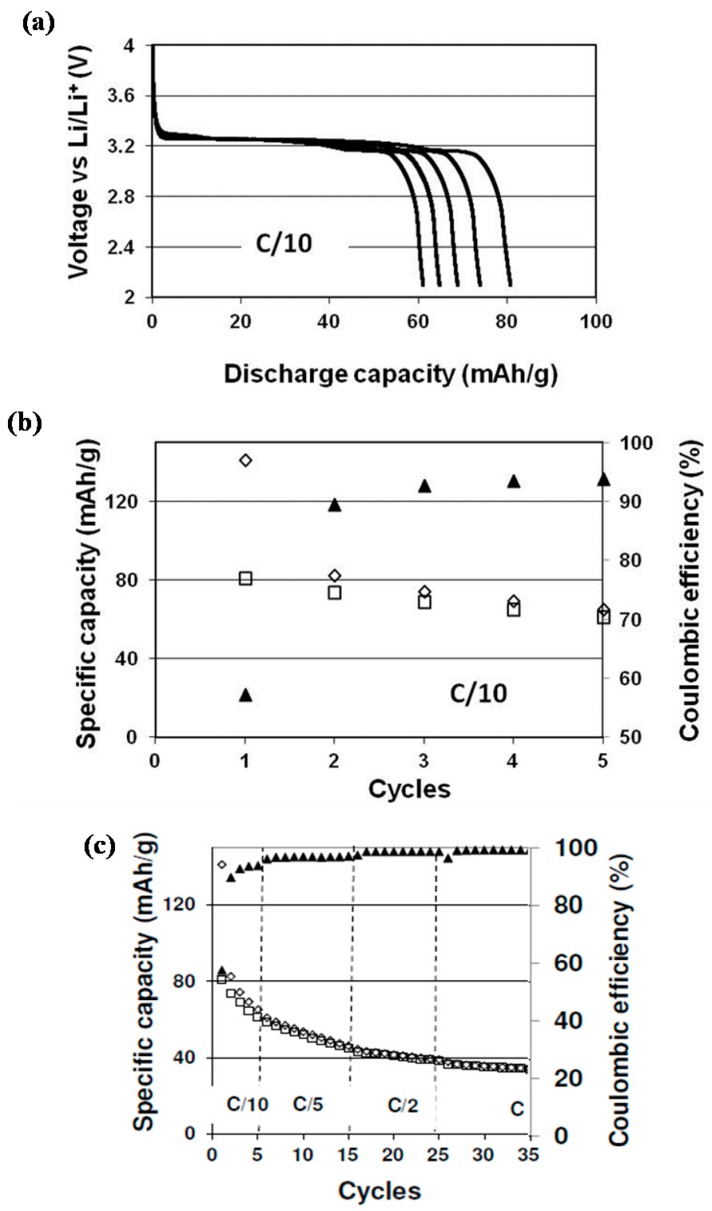
(**a**) Discharge capacity profiles at C/10, (**b**) charge (□) and discharge (◊) capacity and coulombic efficiency (▲) as function of cycles at C/10, (**c**) charge/discharge capacity as a function of the current rate.

Figure 5a shows the discharge profiles at C/10, whereas Figure 5b shows the evolution of the specific capacity and coulombic efficiency of the assembled full battery as a function of cycles at C/10. The capacity values were normalized with respect to the mass of the LFP. Capacities of 140 mAh/g and 80 mAh/g were obtained during the first charge and discharge, respectively. Indeed, the formation of the solid electrolyte interphase (SEI) on the graphite surface induces an irreversible capacity of 60 mAh/g_LFP_; this high capacity loss is in accordance with the irreversible capacity that is obtained on the graphite electrode in a half-cell configuration (Li/graphite based electrode) [13]. A stabilization of the capacity value was observed during the first few cycles, with a discharge capacity of 70 mAh/g after five cycles. The battery exhibits a continuous increase of coulombic efficiency, and attains 95% of coulombic efficiency (CE) at C/10 at the fifth cycle. The absence of graphite SEI layer-forming additives [37], and the presence of residual water, due to the low temperature used during vacuum drying, may explain the weak coulombic efficiency and the discharge capacity loss during cycling. A similar behavior was reported by Leijonmarck et al. [38] for all paper based Li-ion cells; however, he showed that this major drawback due to the highly hygroscopic nature of cellulose can be overcome by increasing the drying temperature up to 170–190 °C, i.e., close to the degradation temperature of pure cellulose.

Despite the progressive cell capacity decay due to a low coulombic efficiency, Figure 5c shows that the increase of the current rate to C/5, C/2, and C induced a drop of ca. 6 mAh/g in the first two cycles, indicating that thick electrodes developed in this study are poorly affected by the current rate.

Work is in progress to optimize the electrolyte composition and the cell drying conditions, in order to improve the cell capacity and cycling stability.

## 4. Conclusions

In summary, this work demonstrates that cellulose nanofibers can be used as a binder to formulate screen printing inks for the fabrication of highly cohesive battery electrodes, and enable a new battery assembling design based on the electrodes front/reverse printing on a paper separator, and the integration of current collectors during the printing stage, thus leading to the fabrication of a fully printed battery in a single unit operation. Cycling tests showed promising results in terms of reversible capacity (80 mAh/g at C/10). Results showed how inks exhibit tailored rheological properties for a screen printing process (shear thinning behavior), and how they can be used to conceive electrochemical devices, such as printed lithium ion batteries. Work is in progress to optimize the electrode mass equilibration that may permit the improvement of the performance. Overall, even if cycling performances are lower than those of conventional cells, the assembling approach presented in this work could be considered a promising and cheaper solution for manufacturing one-step printed lithium ion batteries.

## Figures and Tables

**Table 1:** Physical and electrical properties of the components of the reverse/side-assembled battery.

Sample	Comp.	Loading	Thickness	Porosity	Electrical Conductivity
	Weight (%)	g/m^2^	µm	%	S/m
Negative electrode (GP_CMC_CNF)	(97_2_1)	25 ± 2	34 ± 3	70 ± 3	268 ± 69
Separator/substrate	cellulose	120 ± 1	78 ± 2	47 ± 1	-
Positive electrode (LFP_CB_CMC_CNF)	(70_27_2_1)	70 ± 6	79 ± 7	69 ± 5	37 ± 6

Comp.: composition. One copper current collector is used for the negative electrode; the thickness to the two faces is equal to 68 µm ± 6, 18 µm higher than the copper porous current collector.
